# Adverse Reactions Associated With Cannabis Consumption as Evident From Search Engine Queries

**DOI:** 10.2196/publichealth.8391

**Published:** 2017-10-26

**Authors:** Elad Yom-Tov, Shaul Lev-Ran

**Affiliations:** ^1^ Microsoft Research Herzeliya Israel; ^2^ Lev Hasharon Medical Center Pardesya Israel; ^3^ Sackler Faculty of Medicine Tel-Aviv University Tel-Aviv Israel

**Keywords:** cannabis, search engines, pharmacovigilance

## Abstract

**Background:**

Cannabis is one of the most widely used psychoactive substances worldwide, but adverse drug reactions (ADRs) associated with its use are difficult to study because of its prohibited status in many countries.

**Objective:**

Internet search engine queries have been used to investigate ADRs in pharmaceutical drugs. In this proof-of-concept study, we tested whether these queries can be used to detect the adverse reactions of cannabis use.

**Methods:**

We analyzed anonymized queries from US-based users of Bing, a widely used search engine, made over a period of 6 months and compared the results with the prevalence of cannabis use as reported in the US National Survey on Drug Use in the Household (NSDUH) and with ADRs reported in the Food and Drug Administration’s Adverse Drug Reporting System. Predicted prevalence of cannabis use was estimated from the fraction of people making queries about cannabis, marijuana, and 121 additional synonyms. Predicted ADRs were estimated from queries containing layperson descriptions to 195 ICD-10 symptoms list.

**Results:**

Our results indicated that the predicted prevalence of cannabis use at the US census regional level reaches an *R*^2^ of .71 NSDUH data. Queries for ADRs made by people who also searched for cannabis reveal many of the known adverse effects of cannabis (eg, cough and psychotic symptoms), as well as plausible unknown reactions (eg, pyrexia).

**Conclusions:**

These results indicate that search engine queries can serve as an important tool for the study of adverse reactions of illicit drugs, which are difficult to study in other settings.

## Introduction

Cannabis is the most widely used illicit substance worldwide [1]. The United Nations Office on Drugs and Crime 2017 report indicates that over 180 million people use cannabis annually, accounting for roughly 3.8% of the global population [2], and lifetime prevalence of cannabis use among young adults in the United States has been reported to be around 50% [3].

In recent years, there is an increasing interest from a global health perspective into potential adverse effects of cannabis. This is particularly because of the rapidly shifting landscape regarding the legalization of cannabis in several US states, as well as the rising popularity of medicinal cannabis in several countries worldwide. Several additional factors, such as increased use among adolescents and young adults and the increasing potency of cannabis (as measured by concentration of the principal psychoactive constituent of cannabis, tetrahydrocannabinol [THC]), further contribute to concerns surrounding potential adverse effects of cannabis [4].

Traditionally, the safety of therapeutic agents and adverse effects are studied by a variety of methodologic approaches, including randomized controlled trials, observational studies, and pharmacovigilance studies [5]. Specifically, adverse effects are reported through a variety of regulatory agencies (such as MedWatch by the US Food and Drug Administration [FDA] in the United States and the International Drug Monitoring Programme by the World Health Organization). Several current projects (such as the FDA’s Sentinel Initiative [6], the EU-ADR initiative [7], and the Observational Medical Outcomes Partnership [8]) are beginning to use observational data, including administrative claims and electronic health records, to identify adverse drug reactions (ADRs).

Aside from few cannabinoid-based pharmaceutical drugs, cannabis is largely overlooked by all these methods. The reasons for this oversight are that cannabis is still considered an illicit substance in most countries worldwide, and despite legislative changes in several US states, it is still a Schedule I drug according to federal law in the United States. As use of illicit substances is commonly underreported [9], its use may be associated with social disapproval and stigma, reducing reliable self-report of its use and of associated adverse reactions [10]. Furthermore, as opposed to pharmaceutical drugs, which are tracked by well-established programs described above, illicit drugs are not currently tracked by any such program. It should be noted that though there is no formal definition of *adverse effects* when dealing with illicit drugs, the common FDA definition of “any untoward medical occurrence associated with the use of a drug in humans” [11], with a particular emphasis on undesirable effects of the specific psychoactive substance, remains relevant.

Here we propose to identify the use of cannabis and associated adverse effects through novel observational data, namely, Web search query logs. Search queries contain a cornucopia of world knowledge [12], and prior studies have used query logs to track certain life events [13], the spread of disease [14], and most importantly in this context—adverse effects of medications [15,16]. As such, these data allow analyzing the data from hundreds of millions of people, and in some cases, a significant percentage of the patients using a given drug or an illicit substance.

Accordingly, the aims of this proof-of-concept study are: (1) to provide a *proof of concept* of estimating prevalence of cannabis use and identifying cannabis users through Web search query logs and (2) to explore adverse effects (both prevalence as well as temporality) of cannabis use using Web search query logs. We focus on data from the United States, for both the size of the country and the fact that population-level information on cannabis use exists in this country.

## Methods

### Data

We extracted all queries submitted to the Bing search engine by users located in the United States between November 2016 and April 2017 (inclusive). For each query, we extracted the text entered by the user, time and date, and the state from where the query was issued. Additionally, queries could be grouped to the same user through an anonymized user identifier [17]. We note that Bing users are known to be a representative sample of Internet users in the United States [17].

As baseline data, we extracted the three datasets shown below:

1. Cannabis usage rate (1-year prevalence) per state was extracted from the 2015 National Survey on Drug Use in the Household (NSDUH) survey [18]. This was the most recent available NSDUH state-level data at the time of the study.
2. Usage rate per census region was extracted from the 2012-2014 substate NSDUH estimates [19]. Each region consists of one or more counties. Both the first and second datasets are sponsored by the US Department of Health and Human Services. This was the most recent available NSDUH state-level data at the time of the study.
3. Reports on ADRs to the FDA’s Adverse Drug Reporting System (FAERS) for the years 2013-2016, which mentioned marijuana or cannabis. A total of 11,382 reports from 9218 people were collected.

A list of words possibly related to marijuana consumption, comprising 123 terms, was constructed by browsing Web forums and the Urban Dictionary (see Multimedia Appendix 1).

Queries describing ADRs were identified by testing if they contained one or more of the terms used in previous studies (for a full background, see Yom-Tov and Gabrilovich [5]). This list is of layperson descriptions to 195 ICD-10 symptoms. This list was augmented with the following adverse reactions, listed in FAERS in conjunction with cannabis (“marijuana”) but missing from the list above: emesis, abdominal pain, nausea, drowsiness, red eyes, red conjunctiva, appetite, aggression, agitation, cognitive disorder, delirium, withdrawal, fatigue, gastroschisis, hyperhidrosis, overdose, restlessness, sedation, seizure, and syncope.

Queries that were likely related to news events were removed by excluding queries that had the same text and appeared at a frequency of at least 10,000 times over the data period but with spikes of over 1000 queries during no more than between 1 and 10 days during the data period.

We note that the datasets (ground truth and Bing) do not overlap in dates, which may lead to mismatches in our estimates and hence, lower correlations between estimated and actual use. Therefore, the performance of our models should be considered an underestimate of the possible performance of these models.

### Measures for Analysis of Bing Data

As will be described below, we first found terms (of the list of 123 terms) that are likely associated with cannabis consumption by correlating the fraction of people querying for these terms in each US census region and the cannabis consumption in that census region. We refer to these as the target terms. We then examined the use of terms to describe ADRs in the population using the target terms, compared with the rest of the population of Bing users. Following previous studies [5,15], we employed several ways to measure the association of ADRs with target terms. Here we briefly describe these measures, which give a score to each ADR (for formulas refer to Tables 1 and 2), as follows:

- Query ratio (QR): The fraction of people querying for the ADR who used the target terms, divided by the fraction of people who queried for the ADR (regardless of the target term) ( (f+h)/(e+g) ).
- Query log reaction score (QLRS): This is the original measure developed in Yom-Tov and Gabrilovich [5], which measures the change in queries for the ADR after queries for the target terms. It is computed as the chi-squared score from Table 2.
- Query proportional rate ratio (QPRR): A measure that accounts for the use of a term in the population making target queries, compared with the rest of the population ( d/(d+b) / (c / (a+c) ).
- Proportionality query ratio (PQR): A modification of QLRS found [15] to be more accurate than QLRS in identifying ADRs ( h / (f+h) / (g / (e+g) ).

**Table 1:** A 2×2 table for estimating query proportional rate ratio (QPRR) from Web-based query log data. Letters in the table indicate the number of people in the data who match the relevant conditions.

Conditions	User did not query for target term	User queried for target term
User did not query for ADR^a^	a	b
User queried for ADR	c	d

^a^ADR: adverse drug reaction.

**Table 2:** A 2×2 table for estimating query ratio (QR), proportionality query ratio (PQR), and query log reaction score (QLRS) from Web-based query log data. Letters in the table indicate the number of people in the data who match the relevant conditions.

Conditions	User did not query for target term	User queried for target term
User queried for ADR^a^ after day 0	e	f
User queried for ADR before day 0	g	h

^a^ADR: adverse drug reaction.

We measured the correlation between FAERS reports and Bing data in two ways. First, we selected the 22 ADRs whose prevalence was in the top 95% of FAERS reports for cannabis and assumed these were likely ADRs and that all other ADRs were not associated with cannabis use. We measured the Area Under Curve (AUC) of the Receiver Operating Characteristic Curve for each of the measures derived from the Bing data (see Methods section).

Second, we measured the correlation between the measures computed for Bing data and the number of reports in FAERS for the 85 ADRs that appeared at least once in conjunction with marijuana in FAERS. Following Yom-Tov and Gabrilovich [5], we also used the greedy method used therein for excluding five outliers and showed the improvement in correlation when these are excluded. Outliers (according to Yom-Tov and Gabrilovich [5]) are ADRs that appear with high frequencies in FAERS, but have a low query score, or vice versa. The former happens when ADRs are acute or appear shortly after the substance is used, whereas the latter are ADRs that appear long after people begin using the substance.

## Results

### Correlation With State and Region Prevalence

We filtered the queries to include only those queries that contained one or more words possibly related to cannabis consumption, as detailed in the Methods section. We then calculated the fraction of queries from each state and region using each term.

Region prevalence was modeled using a stepwise linear model [20], where the independent terms are the number of people making queries that mentioned each of the terms in a region, divided by the number of people who queried on Bing from that region. The model reached an *R*^2^ of .71 (n=305 regions), using the terms shown in Table 3, implying that 71% of the variance in the regional prevalence is predictable from the fraction of people making queries shown in Table 3. In this table, a positive slope means that there is a positive correlation between the number of people who use this phrase and the number of people who are known to have used cannabis in the geographic region. Interestingly, the single term “cannabis” reached an *R*^2^ of .24, and the highly collinear term (*r*=.81, *P*<.001) “marijuana” reached an *R*^2^ of .26. The other positive terms in the list reached a lower *R*^2^ (the highest is “caffeine” with *R*^2^ of .18). Therefore, in the next stages of our analysis, where it is important to identify (anonymous) individual people who may have used cannabis, we focus on those people who queried for the terms “cannabis” and “marijuana.”

**Figure 1:**
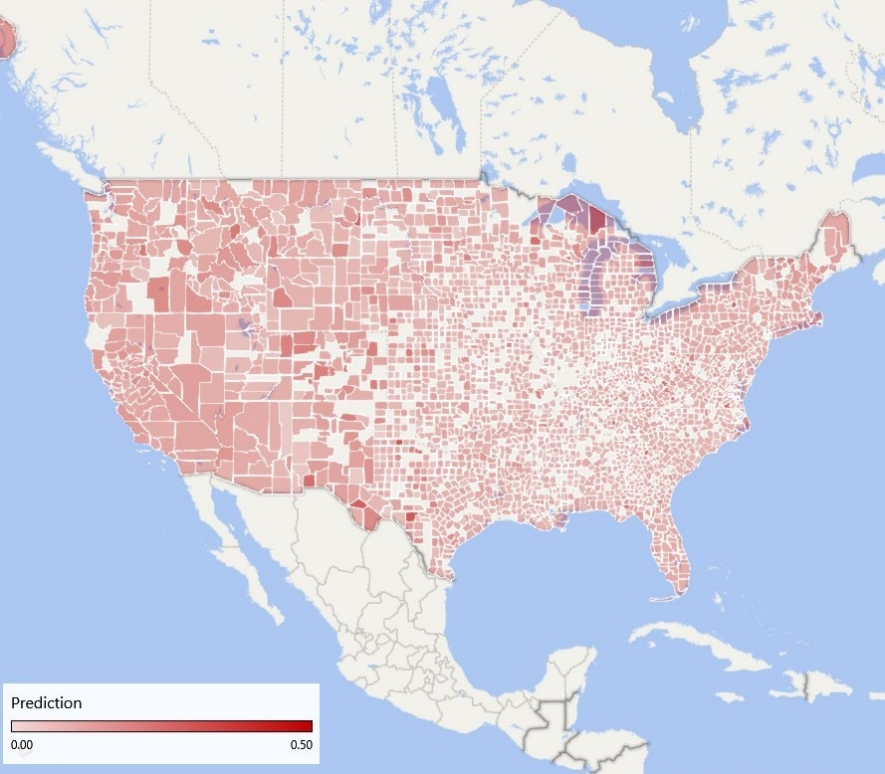
Predicted county-level prevalence of cannabis use based on Web-search queries using terms synonymous with "cannabis" and "marijuana".

Collecting usage data at fine-grained resolution is frequently costly and time-consuming. Therefore, it is important to ascertain whether data that were collected at one (usually course) resolution can be used to build a model that can be applied at other (finer) resolutions. Therefore, we next applied the state-level model to the regional level, so as to estimate the feasibility of using low-resolution ground truth data to estimate higher resolution usage rates. The state-level model reached an *R*^2^ of .93 (n=50). Applying the state-level model to region-level data resulted in a correlation of *r*=.90 (*P*<.001). Applying the region-level model to state-level data resulted in *r*=.57 (*P*<.001). Thus, it is possible to apply a model created from one level of aggregation to another level of aggregation, with a reasonably small degradation in performance. Therefore, we applied the region-level model to county-level data. The predicted prevalence of cannabis use at a county level is shown in Figure 1.

### Correlation With FAERS Reports

The AUCs and correlations for the four measures (QR, QLRS, PQRR, and PQR) are shown in Table 4. Since the QR and QLRS measures achieved similar correlations and AUCs, we focused on the QLRS measure, which has also been validated for pharmaceutical drugs [5,15].

**Figure 2:**
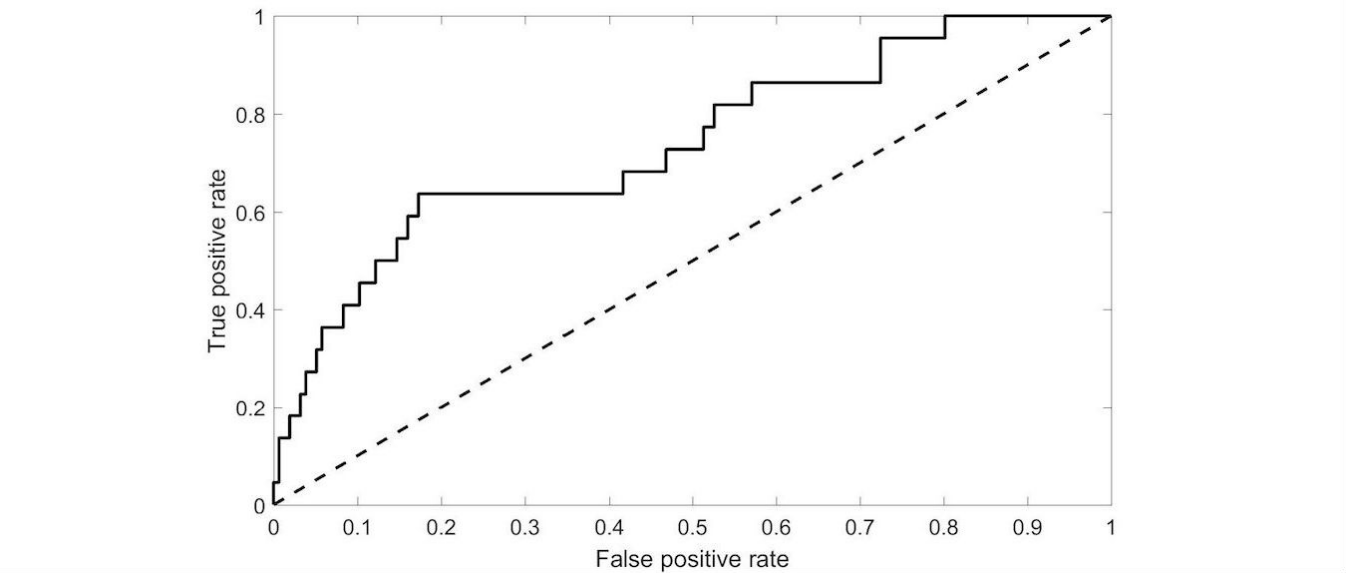
Receiving Operating Curve (ROC) analysis for detecting Adverse Drug Reactions appearing in Food and Drug Administration's Adverse Drug Reporting System (FAERS) using Query Log Reaction Score (QLRS).

The AUC for QLRS is shown in Figure 2. As the figure shows, QLRS is especially useful at detecting ADRs with a high likelihood to be of relevance. The ADRs rated highest in QLRS were in descending order:

1. anxiety
2. pain
3. overdose
4. paranoia
5. depression
6. withdrawal
7. seizure
8. hallucination
9. headache
10. cough

**Table 3:** Statistically significant terms in a stepwise linear model to predict US region incidence of cannabis use.

Term	Slope direction^a^
Antisocial behavior	−
Attention deficit	+
Blue	+
Bozo	+
Caffeine	+
Cannabis	−
Color	−
Domes	−
Hombre	−
Mikes	+
Peyote	−
Psychiatry	+
Speedball	+
Spoon	−
Stuff	−
Tickets	−
Valium	+
Draw	+
Jay	+
Marijuana	+

^a^Positive slope means that there is a positive correlation between the number of people who use this phrase and the number of people who are known to have used cannabis in the geographic region.

**Table 4:** Area Under the Receiving Operating Curve and Spearman correlation between Food and Drug Administration’s Adverse Drug Reporting System reports and query measures for terms synonymous with “cannabis” and “marijuana.”

Measure	AUC^a^	Correlation
QR^b^	0.77	.39
QLRS^c^	0.74	.31
QPRR^d^	0.68	.35
PQR^e^	0.61	.27

^a^AUC: area under curve.

^b^QR: query ratio.

^c^QLRS: query log reaction score.

^d^QPRR: query proportional rate ratio.

^e^PQR: proportionality query ratio.

**Figure 3:**
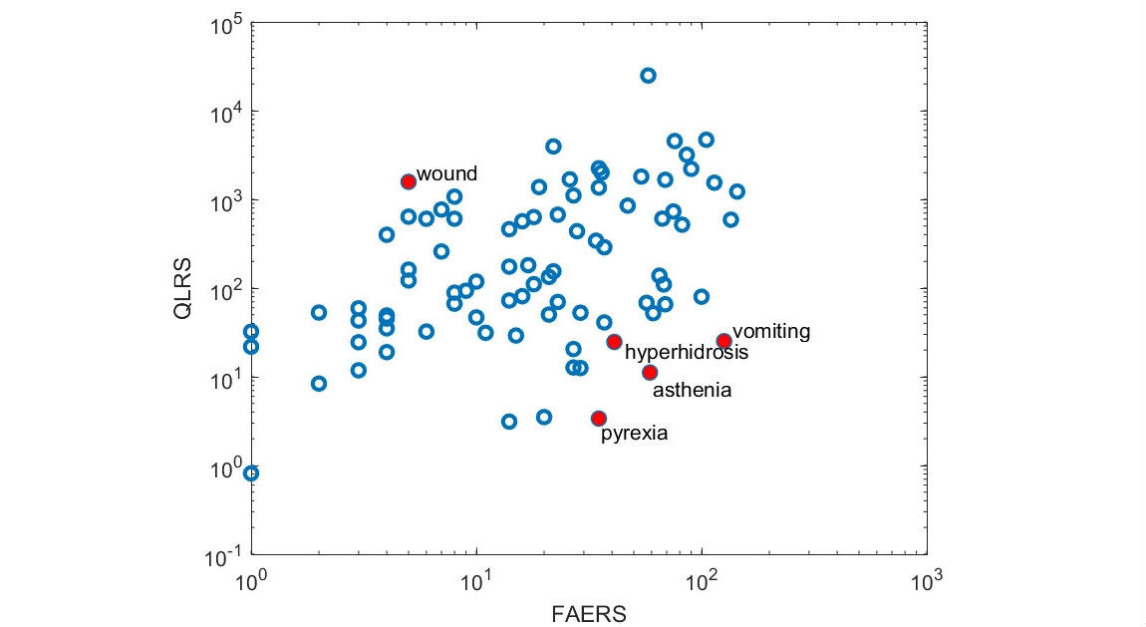
Plotted Query Log Reaction Scores (QLRS) versus the number of reports in Food and Drug Administration's Adverse Drug Reporting System (FAERS). Each dot represents an adverse drug reaction. Axes are log-scaled. Full red dots denote outliers, as identified by the analysis. Correlation between FAERS counts and QLRS scores for the blue unfilled dots is .42 (*P*<.001).

### Outliers

Figure 3 shows a scatter plot of the QLRS score versus the number of reports in FAERS, for the 85 ADRs analyzed. Marked separately are the ADRs identified as outliers using an iterative removal process, as described in Yom-Tov and Gabrilovich [5]. Correlation between FAERS counts and QLRS scores for the blue unfilled dots is .42 (*P*<.001), compared with .31 when these are not removed.

### Temporal Profiles of Symptom Queries

We assessed the temporal patterns of the ADRs rated highest in QLRS (see above) by calculating the fraction of queries that mentioned an ADR, compared with the fraction of all ADR queries per day [13], as a function of the number of days since the first query for “cannabis” or “marijuana” by each person.

**Figure 4:**
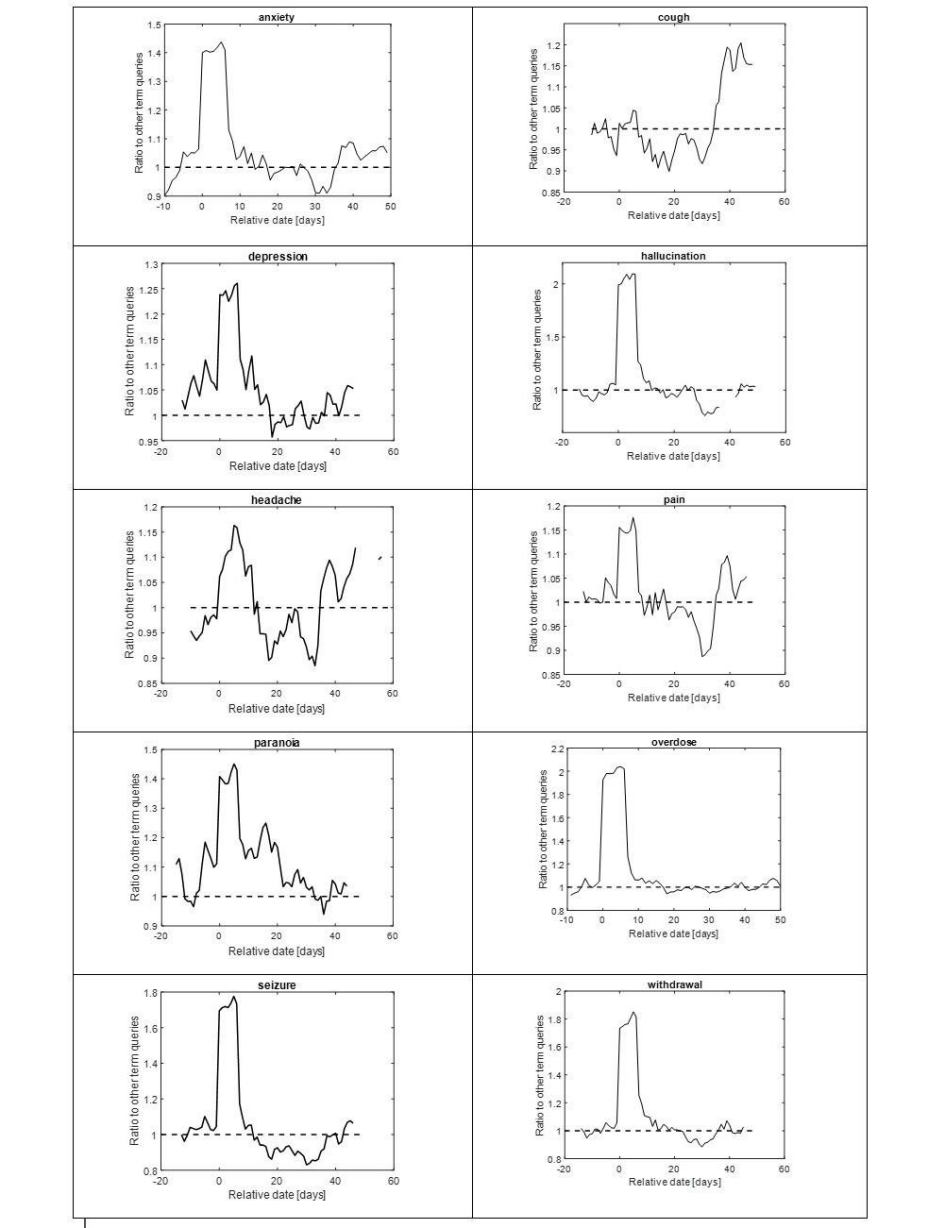
Temporal profiles of symptom queries (Day 0=first query for terms synonymous with "cannabis" and "marijuana"). Time series are smoothed with a 7-day moving average window. Shown are days with the 25% highest activity.

The resulting patterns are shown in Figure 4. As the figure shows, most ADRs (anxiety, depression, hallucination, pain, overdose, seizure, and withdrawal) begin on day 0 (the day on which the first query for “cannabis” or “marijuana” was made) and drop to baseline level within the following 10 days. However, “headache” begins only 3 to 5 days after day 0, and “cough” rises after approximately 40 days.

## Discussion

### Principal Findings

In this study, we sought to explore the applicability of Web search data for studying the prevalence of cannabis use as well as potential adverse effects thereof. Using a well-established model that has been repeatedly shown to be effective in exploring ADRs of pharmaceutical agents, we show that this novel low-cost method: (1) provides estimate data which is in line with epidemiological-derived studies on the spatial distribution of cannabis use and (2) reveals less common adverse effects of cannabis that are largely unreported. Together, this serves as a proof-of-concept for using this type of research design for studying the adverse effects of illicit drugs.

Our results from state and region-based data when compared with survey-based data indicate that it is possible to apply a model created from one level of aggregation to another, with small degradation in performance. Accordingly, we can estimate cannabis usage at the county level. The high accuracy of the model fit (which is similar in value to models for pharmaceutical drug use [5]) may indicate that: (1) people who use cannabis (particularly those concerned about adverse effects) ask about it online, perhaps because it is an anonymous channel of communication, which is thus more accessible and less stigmatizing than “official” channels such as family physicians and (2) that it is possible to estimate ADRs from these data. This may have significant implications for public health, as county-level data concerning drug use and other highly stigmatized behaviors are scarce and usually nonexistent. Estimates of county-level use may allow tailoring interventions in local educational and community-based facilities, focusing on specific counties within a region with highest rates of use.

Comparing the appearance of frequent ADRs in FAERS and QLRS, our results show high rates of correlation. Furthermore, several of the common side effects found in Web searches have been repeatedly reported in clinical and epidemiological studies. Our findings on common reports of anxiety and depression-related symptoms are in line with previous reports based on conventional data collection [21]. We found high rates of searches associated with cough among cannabis users; the findings echo research indicating higher rates of symptoms of chronic bronchitis compared with nonusers [22]. Common searches for psychotic symptoms such as paranoia and hallucinations are in line with previous reports of cannabis intoxication [23] as well as long-term effects of cannabis [24]. Other ADRs related to intensity of use (overdose) and cessation of cannabis use (withdrawal) echo previous reports as well [25]. Alongside these commonly reported adverse effects, specific pain-related ADRs (eg, pain and headache) and “seizures” found in our Web-based results are largely underreported and possibly understudied. We note that, since QLRS accounts for the time of ADR query vis-à-vis the query for cannabis, queries for pain occur after those for cannabis, and therefore, it is not the case that pain is the cause for queries regarding cannabis but more likely the obverse.

Alhough the correlation of ADRs as per FAERS and QLRS is high, there are several outliers: hyperhidrosis, asthenia, pyrexia, and vomiting appeared more commonly in FAERS compared with QLRS. This may indicate more acute side effects. For example, hyperhidrosis and asthenia may indicate panic-related symptoms, which may appear acutely following cannabis use [26]. Although cannabis has been shown to reduce (not increase) body temperature in preclinical models [27], there are reports of individuals reporting a subjective feeling of warmth when intoxicated [28], which may have increased searches of “pyrexia” and synonymous terms.

Our results regarding the temporal appearance of ADRs reveal interesting findings. For several potential ADRs, individuals searched for them on the same day on which the first query for “cannabis” or “marijuana” was made (“day 0”). Of these, some represent potentially acute ADRs (eg, hallucinations and overdose), whereas some may represent an inverse relationship. For example, in the case of seizure and pain, it is possible that individuals seeking relief from these problems conducted searches for cannabis as a potential treatment. However, as stated above, QLRS takes the time of query for ADR relative to that of cannabis into account. Therefore, we hypothesize that these queries were possibly caused by the ineffectiveness of cannabis for these symptoms, which caused people to continue asking about them (and even increasing the number of queries for them) after querying for cannabis. This could not be directly explored in this study. Interestingly, though anxiety and depression have been reported (in some cohorts) as long-term ADRs associated with cannabis, these appeared on “day 0” of the cannabis search as per QLRS.

### Limitations

The main drawback of relying on Web search data is that it is inherently noisy. It is often impossible to ascertain whether a person searching for drugs and ADRs is doing so out of curiosity or conducting research for himself, a relative, or even for a patient. Admittedly, Internet users comprise a biased sample of the population, and thus the ADRs discovered may not be fully representative of the entire population. Nonetheless, our results suggest that the sheer size of the data alleviates these concerns, and the proposed method is able to identify adverse effects of drugs that are not captured by existing surveillance mechanisms. Another limitation of this study is using a restricted set of symptoms expanded through the use of synonyms. Although a larger dictionary would have allowed identification of additional (and possibly rarer) ADRs, our focus on more common symptoms is likely to lead to better identification of the more common concerns to patients. Future work will focus on professionally used term dictionaries which will allow focusing on knowledgeable patients and health providers. Another way to strengthen our results is the use of non-English search data, which will increase the volume of data (and the size of the observed population), thus enabling the analysis of less frequent drugs and ADRs. In any case, a particular challenge when exploring ADRs of illicit drugs is the plethora of street-names that may evolve rapidly and differ substantially across regions and countries. In addition, this approach raises specific challenges when exploring long-term effects of misuse of prescription drugs (such as opioids, stimulants, and sedatives), as this requires differentiating cases of prescription medication use (ie, according to physicians’ recommendations) and misuse (eg, abuse or dependence). Finally, although this work is based on data from a large Internet search engine, it does not cover the entire population. However, privacy concerns preclude conducting our analysis across search engines, as the latter never share information about their users. Nevertheless, given the sheer number of users whose data were analyzed in the study (33% of the US population, which is especially notable compared with most epidemiological studies), we believe our findings are novel and significant. It should also be emphasized that QLRS discovers ADRs via aggregating queries across multiple users and query sessions. Consequently, the output of our method does not include any private, personal, or user-specific data whatsoever.

### Conclusions

With rising prevalence rates in recent years and a growing controversy on its health-related effects and legal status, cannabis use is widely debated in academic, legislative, and popular platforms. In light of this debate, long-term effects of cannabis use must be carefully explored. Current epidemiological research, in the form of face-to-face interviews or telephone screening, suffers from several methodological drawbacks, including, for example, limited sample size and report bias. The latter may be particularly important when exploring effects of illicit substances, as false reporting is common because of social desirability bias [29]. Our proposed method provides a novel, low-cost, and rapid method for exploring prevalence of use, characteristics of users, and underreported adverse effects of illicit drug use. To the best of our knowledge, these methods have not been reported before and may provide a particularly valuable method for studying use and effects of illicit drugs.

## Appendix

Multimedia Appendix 1 Terms used to identify marijuana use.

## Abbreviations

ADR: adverse drug reactions
AUC: Area Under Curve
FAERS: Food and Drug Administration’s Adverse Drug Reporting System
FDA: Food and Drug Administration
NSDUH: National Survey on Drug Use in the Household
PQR: proportionality query ratio
QLRS: query log reaction score
QPRR: query proportional rate ratio
QR: query ratio
