# Supplementary material for: Adverse Reactions Associated With Cannabis Consumption as Evident From Search Engine Queries
Source: JMIR Public Health Surveill. 2017 Oct 26;3(4):e77. doi: 10.2196/publichealth.8391 (PMC5680525; doi:10.2196/publichealth.8391)
Supplement: Multimedia Appendix 1 [file publichealth_v3i4e77_app1.pdf]

Multimedia Appendix A: Terms used to identify marijuana use

420

bammer

BC bud

Bhang

blunt

bomb

bone

boo

boo-yah

bubonic chronic

bud

buddha

burger

Cali

candle

cheeba

chiba

chief

chiefs

chino

choke

chronic

cigga-weed

cigweed

clickem

colitas

combustible herbage

crippy

cronick

dank

dirt weed

ditch weed

doja

dolja

doob

doober

doobie

dope

drat

draw

dro

dub

dube

Dutchie

endo

erve

fatty

fatty boom blatty

gange

ganja

ganje

giggle stick

gonj

grass

green

green bud

hashish

hasheesh

heim

herb

herbal refreshment

hippie lettuce

hog leg

hooter

hydro

indica

indo

j

jay

jib

joint

keef

kief

kill

kind bud

la la

left-handed cigarette

limbo

loud

marijuana

marihuana

Mary Jane

Mexican dirt weed

mighty mez

MJ

mota

Mr. J

nib

nugget

onion

paca lolo

pakaloco

pakalolo

pato

pot

purp

reefer

reggie

reggs

roach

schwag

shake

shwag

skater

skunk weed

smoke

spliff

spliffy

sticky icky icky

sweet

tea

Thai stick

tical

toke

treats

trees

tunechi

tweed

wacky tobaccy

weed

whifty

woolies

zombie
